# Assessment of Cellular Uptake Efficiency According to Multiple Inhibitors of Fe_3_O_4_-Au Core-Shell Nanoparticles: Possibility to Control Specific Endocytosis in Colorectal Cancer Cells

**DOI:** 10.1186/s11671-020-03395-w

**Published:** 2020-08-17

**Authors:** Bo Gi Park, Yu Jin Kim, Ji Hyun Min, Taek-Chin Cheong, Sang Hwan Nam, Nam-Hyuk Cho, Young Keun Kim, Kyu Back Lee

**Affiliations:** 1 Department of Biomedical Engineering, College of Health Science, Korea University, Seoul, 02841 South Korea; 2 Institute for High Technology Materials and Devices, College of Engineering, Korea University, Seoul, 02841 South Korea; 3 Department of Materials Science and Engineering, College of Engineering, Korea University, Seoul, 02841 South Korea; 4 Department of Microbiology and Immunology, College of Medicine, Seoul National University, Seoul, 03080 South Korea; 5 Center for Convergent Research of Emerging Virus Infection, Korea Research Institute of Chemical Technology, Daejeon, 34114 South Korea

**Keywords:** Fe_3_O_4_-Au core-shell NPs, Receptor-mediated endocytosis, Muc1, Cancer targeting

## Abstract

Magnetite (Fe_3_O_4_)-gold (Au) core-shell nanoparticles (NPs) have unique magnetic and optical properties. When combined with biological moieties, these NPs can offer new strategies for biomedical applications, such as drug delivery and cancer targeting. Here, we present an effective method for the controllable cellular uptake of magnetic core-shell NP systems combined with biological moieties. Vimentin, which is the structural protein, has been biochemically confirmed to affect phagocytosis potently. In addition, vimentin affects exogenic materials internalization into cells even though under multiple inhibitions of biological moieties. In this study, we demonstrate the cellular internalization performance of Fe_3_O_4_-Au core-shell NPs with surface modification using a combination of biological moieties. The photofluorescence of vimentin-tagged NPs remained unaffected under multiple inhibition tests, indicating that the NPs were minimally influenced by nystatin, dynasore, cytochalasin D, and even the Muc1 antibody (Ab). Consequently, this result indicates that the Muc1 Ab can target specific molecules and can control specific endocytosis. Besides, we show the possibility of controlling specific endocytosis in colorectal cancer cells.

## Introduction

Nanomaterials have opened new avenues for clinical diagnostics and therapeutics. Especially, nanoparticles (NPs) are one of the most important tools and have been used in applications such as biosensors [1, 2], diagnostics [3, 4], and targeted drug delivery systems [5, 6]. For biomedical applications, NPs generally composed of organic materials surrounding surface on core materials [7–9]. The core materials, which is consisted of magnetic materials, semiconductor materials, or other types of materials, have useful physicochemical properties, and the outer organic surface provides chemical stability and functionality to the NPs. For applications in biological targeting systems, not only the physicochemical properties but also the outer organic surface, which are biofunctionalized for targeting, are critical parameters. Examples of targeting moieties for functionalization are antibodies or ligands that are specific for a target. Depending on the biofunctionalized materials on the outer surface, the endocytosis mechanisms of NPs are determined. The mechanism that allows NPs to enter cells has been the subject of many recent research works because of their importance in nanomedicine applications [10–15].

In particular, magnetic NPs have been widely used in many specific site targeting applications, including cell sorting [16, 17], MRI [18], DNA isolation [19], drug delivery [20], hyperthermia treatment [21], and cancer targeting [22]. Among various magnetic NPs, magnetite nanocrystals have been most widely used in biomedical applications because of their biocompatibility and chemical stability. Although many efforts have been devoted to biomedical applications using magnetic NPs, there still are some critical issues such as good dispersibility in aqueous solution, functionality, and biocompatibility. To overcome these problems, many studies have focused on the surface modification of NPs using a variety of functional groups (e.g., carboxyl and amine groups) [23]. However, the attachment of the functional groups to the surface of magnetite NPs is a time-consuming and laborious process. Given this fact, core-shell-type Au-coated magnetic NPs are attractive because the Au surface can link easily to biomolecules and organic materials.

Especially, the magnetic properties of the magnetic core-Au shell NPs enable the magnetic separation, increase resolution in MRI imaging, and can be applied to hyperthermia therapy. Moreover, the superior chemical binding properties of gold are advantageous for building receptor-mediated delivery systems for specific cancer-targeting [24–26].

Over the past few decades, many researchers have reported receptor-mediated delivery systems for cancer targeting [27–29].

Receptor-mediated targeting of cancer cells is a form of active targeting. The choice of target is the key for effective active targeting, and the targets must be overexpressed on the extracellular membrane. Most researchers have used monoclonal antibodies for cancer treatment, and the therapeutic effect could be greatly increased when monoclonal antibody therapies are combined with conventional chemotherapy [30]. Despite the success of monoclonal antibody therapy, monoclonal antibodies present several limitations in cancer targeting. Their large size (approximately 150 kDa) is a major obstacle for tumor penetration [31, 32], and their low stability and low solubility hinder their widespread use [33]. The inhomogeneous direction of their attachment on the targeting carrier is also considered an obstacle to nonspecific binding. To produce antibodies with improved tumor penetration, a wide range of antibody formats have been engineered and tested [34]. Apart from classical antibodies, a unique antibody format is present in species from the family Camelidae. The so-called heavy-chain antibodies (HCAbs) occur naturally in the peripheral blood and milk of these species. The antigen-binding fragments of such HCAbs are composed of one single domain, the heavy-chain variable domain (VH) of the camelid HCAb (VHH). The VHH, recombinantly obtained after cloning and expression in bacteria or fungi, is called a nanobody. It has a molecular weight of 11–15 kDa and is the smallest antibody among all mAbs [35–37]. Not only their small size makes them potentially suitable as targeting probes against antigens in isolated locations, but also their easily modifiable terminal end is attractive for application in cancer targeting.

The efficient delivery of NPs with suitable targeting and internalization of cells are also important factors in the delivery system. It has been reported that vimentin acts an important role as a component of pathogen attachment and intracellular entry pathways. Silencing of vimentin gene expression inhibits phagocytosis [38], whereas cleaved vimentin is a signal that significantly increases phagocytosis [39]. Therefore, neutralizing cell phagocytosis resistance caused by vimentin on the cell surface is important for efficient nanoparticle delivery.

In this study, we investigate the endocytosis pathways of nanobody-tagged Fe_3_O_4_-Au core-shell NPs modified with PEG (polyethylene glycol) spacers with different lengths. Vimentin, which is known to have a strong effect on phagocytosis by biochemical experiments [39], was compared as a control, and it was confirmed that it effectively acts on cell internalization of NPs. Besides, the Muc1, which is a cell surface glycoprotein and overexpressed in various cancer, such as pancreatic, breast, lung, and stomach cancer, is utilized as a cancer-targeting biomarker. We confirmed the efficient internalization of Fe_3_O_4_Au core-shell NPs and the methods of controllable targeting to cancer cells through the Muc1 receptor-mediated endocytosis pathway in colonic cells.

## Materials and Methods

### Materials

Gold (III) acetate (Au(OOCCH3)3, 99.9%) was obtained from Alfa Aesar. Other chemicals including iron (III) acetylacetonate (Fe(acac)_3_, 99.9%), 1,2-hexadecanediol (C14H29CH(OH)CH2(OH), 90%), poly(ethylene glycol)-block-poly(propylene glycol)-block-poly(ethylene glycol) (PEG-PPG-PEG), and octyl ether (C8H17OC8H17, 99%) were purchased from Sigma-Aldrich and used as received. Alpha-pyridyl-2-disulfid-omega-carboxy succinimidyl ester poly(ethylene glycol) (OPSS-PEG-NHS) (2K, 5K, and 10K) was purchased from Nanocs. Sodium bicarbonate, WST-1, chlorpromazine, nystatin, cytochalasin D, dynasore, brefeldin A (BFA), monensin, and trypan blue were purchased from Sigma-Aldrich. Cy3 and Cy7.5 were purchased from Lumiprobe. Anti-Muc1 Ab was purchased from Abcam Inc. (Cambridge, MA). Phosphate-buffered saline (PBS), Dulbecco’s modified Eagle’s medium, and fetal bovine serum were purchased from Invitrogen Corp.

### Synthesis of Fe_3_O_4_-Au Core-Shell NPs

The Fe_3_O_4_-Au core-shell NPs were synthesized via a nanoemulsion method. The synthetic process for core-shell NPs consists of two steps: (1) formation of the Fe_3_O_4_ core NPs and (2) coating of the Au shell on the magnetic NPs. In the first step, the Fe_3_O_4_ NPs were prepared from a mixed solution of Fe(acac)_3_ (0.1766 g or 0.5 mmol), 1,2-hexadecandiol (0.6468 g or 2.5 mmol), and block copolymer (poly(ethylene oxide)-poly(propylene oxide)-poly(ethylene oxide); PEO-PPO-PEO) (0.4 ~ 1.2 g) in octyl ether. The mixed solution was heated at 300 °C to reduce the Fe precursor. The formation of Fe_3_O_4_ core NPs was completed by cooling the heated solution. The second process was continuously conducted without any purification process after the formation of the magnetic core. Au precursors (0.2338 g or 0.62 mmol) and 1,2-hexadecandiol (0.88 g, 3.4 mmol) were added into the emulsion consisting of Fe_3_O_4_ NPs, and then the mixed solution was heated at 230 °C. After cooling down to room temperature, the emulsion was precipitated by centrifugation and the core-shell NPs were separated.

### Construction of Recombinant Anti-Muc1-VHH 5-24 K10 Expression Vector

Polymerase chain reaction (PCR) was performed using the forward primer 5′-CCGAATTCGCCGATGTGCAGCTGACCGAG-3′ and the reverse primer 5′-CGG CTCGAGCTTCTTCTTCTTCTTCTTCTTCTTCTTCTTGCCTGAGGAGACGGTGACCTG-3′. The PCR product was digested with EcoRI and XhoI and gel-purified using the QIA quick Gel Extraction Kit (QIAGEN, Valencia, CA, USA). The purified PCR product was cloned into EcoRI/XhoI-digested pET-23a (Novagen, Darmstadt, Germany). *Escherichia coli* (*E. coli*) DH5α (RBC Bioscience, Xindian, Taiwan) was transformed with the resulting construct by heat shock and selected on LB agar plates containing 100 μg/mL ampicillin (Duchefa Biochemie, Haarlem, The Netherlands).

### Expression and Purification of Recombinant Protein

To express and purify recombinant anti-Muc1-VHH 5-24 K10 protein, *E. coli* BL21 strains (RBC Bioscience, Xindian, Taiwan) were transformed with pET-23a-anti-Muc1-VHH 5-24 K10. Bacteria were then grown in LB broth containing ampicillin (100 μg/mL). Protein expression was induced by isopropyl β-d-thiogalactoside (IPTG) (Duchefa Biochemie, Haarlem, The Netherlands) at a final concentration of 0.4 mM for 5 h at 37 °C. Bacterial pellets were resuspended in lysis buffer (50 mM NaH_2_PO_4_, pH 8.0; 300 mM NaCl) followed by sonication on ice for 10 min. Sonicated lysates were centrifuged at 20,000×*g* for 20 min at 4 °C and subjected to Ni-NTA His·Bind Resin (Peptron, Daejeon, Korea). His-tagged proteins that were bound to the resin were eluted with elution buffer (50 mM NaH_2_PO_4_, pH 8.0; 300 mM NaCl; 150 mM imidazole). Purified protein was separated on 15% SDS-PAGE gel.

### Modification of Core-Shell Fe_3_O_4_-Au NPs

OPSS-PEG-NHS at various lengths (2, 5, and 10 K) was dissolved in 0.1 M sodium bicarbonate for activation of the thiol groups. Activated OPSS-PEG-NHS was added to the solution of synthesized core-shell Fe_3_O_4_-Au NPs and agitated for 12 h at 4 °C. The thiol groups of activated OPSS-PEG-NHS were covalently linked to the Au surface of the core-shell NPs. Then, a nanobody solution (0.25 mg/mL) was added to the PEGylated Fe_3_O_4_-Au core-shell NPs for 12 h at 4 °C. The amine groups of the ten lysine (K) tails at the terminal were covalently linked to the NHS groups of OPSS-PEG-NHS at pH 8.3. Cy3 and Cy7.5 were tagged to the residual amine groups of the nanobody.

### Internalization Curve

CT26 cells were seeded at 5 × 10^3^ cells per well in a clear-bottom 96-well plate and incubated in 250 μL of culture medium for 24 h at 37 °C in 5% CO_2_ in the dark. The medium was removed, and 250 μL of fresh culture medium containing 50 μg/mL Cy3-labeled Fe_3_O_4_-Au NPs and PEG-Cy3 or PEG-nanobody-Cy3-labeled NPs were added to each well. The cells were further incubated for different periods (0, 10, 20, 30, 60, 120, and 360 min). The cells were then washed three times with PBS to remove free NPs, and the fluorescence of each well was measured with trypan blue as a membrane-impermeable fluorescence quencher by SpectraMAX GEMINI (Molecular Devices, CA, USA). Each experiment was carried out with equal amounts of NPs (50 μg/mL) and repeated three times [40].

### Inhibition Test

CT26 cells were seeded at 5 × 10^3^ cells per well in a clear-bottom 96-well plate and incubated in 250 μL of medium for 24 h at 37 °C in 5% CO_2_ in the dark. The medium was removed and 250 μL of fresh culture medium containing either 20 μg/mL chlorpromazine (CPZ), 50 μg/mL nystatin, 20 μg/mL cytochalasin D, 25 μg/mL dynasore, 20 μg/mL BFA, 140 μg/mL monensin, or 5 μM anti-Muc1 Ab were added, and the cells were incubated for 1 h.

The medium was removed again, and 250 μL of culture medium containing 50 μg/mL Cy3-labeled Fe_3_O_4_-Au NPs, PEG-Cy3-labeled NPs, PEG-nanobody-Cy3-labeled NPs, or vimentin-Cy3-labeled NPs was added.

After 1 h at 37 °C and 5% CO_2_, the cells were washed three times with PBS to remove free NPs, and the fluorescence was measured with trypan blue as a membrane-impermeable fluorescence quencher by SpectraMAX GEMINI (Molecular Devices, CA, USA). Each experiment was carried out with equal amounts of NPs (50 μg/mL) and repeated four times.

## Results and Discussion

**Fig. 1:**
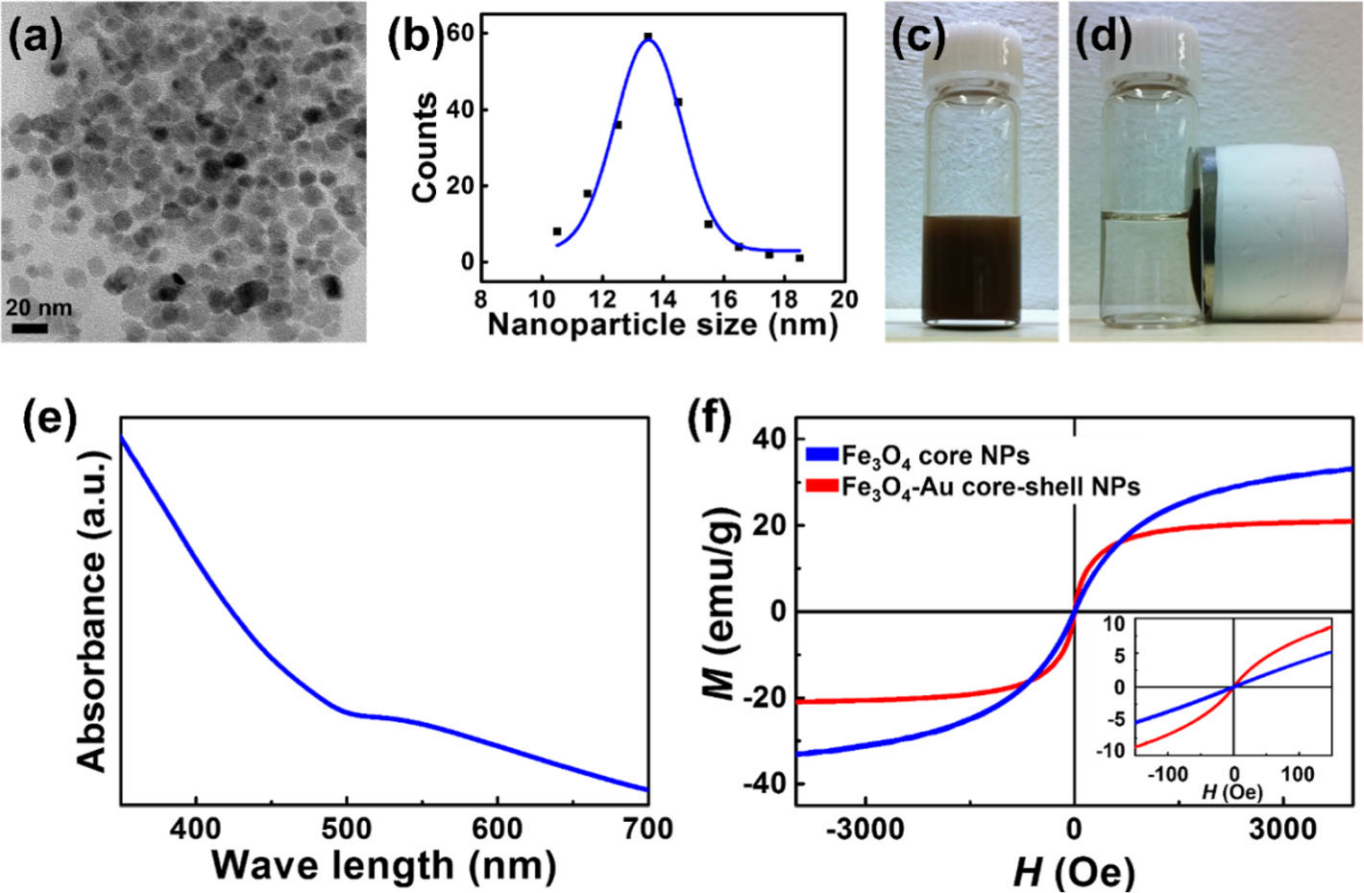
Characterization of the synthesized Fe_3_O_4_-Au core-shell NPs. **a**, **b** TEM observations of the synthesized Fe_3_O_4_-Au core-shell NPs. **c**, **d** NPs in aqueous solution, before and after applying an external magnetic field. **e** UV absorbance peak of synthesized core-shell NPs appears at ~ 530 nm. **f** Magnetic hysteresis loops of Fe_3_O_4_ core

The core-shell NPs were synthesized by a published method [16, 17]. Transmission electron microscopy (TEM) observations in Fig. 1a, b show that the Fe_3_O_4_-Au core-shell NPs were spherical with an average diameter of 13.5 nm and narrow size distribution.

The increase from the ~ 8.5 nm of the core NP (Fe_3_O_4_) stems from the coating of ~ 2.5-nm-thick Au shell on the core surface, resulting in a core-shell NP. A high-resolution TEM image with the fast Fourier transform (FFT) analysis of the Fe_3_O_4_-Au core-shell NP is included in Supplementary Information Fig. S1.

The product manufactured in organic solvent was purified using magnetic separation and transferred into the water.

The core-shell NPs were well dispersed and stable in water without any surface modification, owing to residual block co-polymers that were present on the NPs.

Figure 1c and d show the NPs in aqueous solution before and after applying an external magnetic field. Under an external magnetic field, the core-shell NPs rapidly changed from a homogeneous dispersion (Fig. 1c) to a clear and transparent solution (Fig. 1d).

The absorption band of the core-shell NPs was investigated using UV-Vis spectrometry. As shown in Fig. 1e, an absorbance peak appeared at ~ 530 nm, indicating the presence of Au on the surface of the NPs (Supplementary Information Fig. S2 includes the result of EDX data for Fe_3_O_4_-Au core-shell NPs). As the sample had been purified, the optical results demonstrated the formation of the core-shell structure.

Magnetic hysteresis loops were obtained from vibrating sample measurements to investigate the magnetic properties of the Fe_3_O_4_ core and the core-shell NPs. Both NPs showed superparamagnetic behavior with a coercivity of near 0 Oe at room temperature (Fig. 1f).

As reported in previous works, the susceptibility of core-shell NPs was higher than that of the magnetite NPs, which could be partly owing to proximity effects and unique spatial configurations [41, 42]. Besides, the saturation magnetizations of the core NPs and the core-shell NPs are ~ 37 emu/g and ~ 21 emu/g at 10 kOe, respectively. The difference in the Ms stems from the existence of a nonmagnetic component (Au) in the core-shell NPs.

**Fig. 2:**
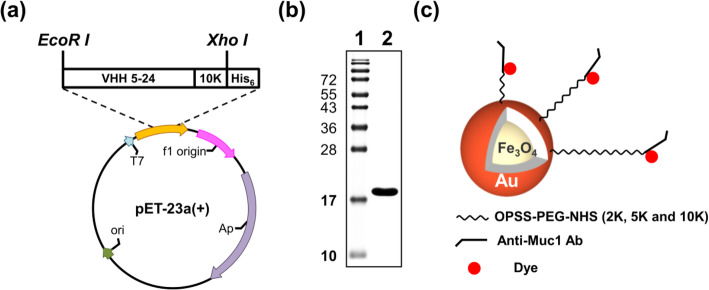
Expression and purification of anti-Muc1-VHH 5-24 K10 fusion protein. The VHH 5-24 K10 gene was cloned in-frame to produce **a** pET-23a-anti-Muc1-VHH 5-24 K10 after **b** purification of Muc1-VHH 5-24 K10 fusion protein. Purified protein was separated on 15% SDS-PAGE. Lane 1 protein ladder. Lane 2 purified protein. **c** Schematic illustration of PEG-nanobody-dye-labeled NPs used in this study

The VHH 5-24 K10 gene was cloned in-frame to produce pET-23a-anti-Muc1-VHH 5-24 K10 after PCR amplification (Fig. 2a). The recombinant protein was expressed in *E. coli* BL21 that was transformed with pET-23a-anti-Muc1-VHH 5-24 K10 after induction with IPTG and purified by Ni-NTA His·Bind Resin. Recombinant anti-Muc1-VHH 5-24 K10 was readily expressed in *E. coli* as a soluble 18-kDa protein. From a 1-L culture, we obtained 1 ± 0.5 mg of purified recombinant anti-Muc1-VHH 5-24 K10.

The purified protein was verified by 15% SDS-PAGE gel. Coomassie blue staining of the purified protein revealed that it was > 95% pure (Fig. 2b). The synthesized Fe_3_O_4_-Au NPs were modified in three steps, namely PEGylation, antibody tagging, and dye labeling (Fig. 2c). After each modification step, the zeta potential was measured to confirm the successful modification. Table 1 shows the effect of modification on the corresponding zeta potentials. The zeta potential of bare core-shell NPs was − 19.8 ± 6.68 mV. PEGylation of NPs was carried out using OPSS-PEG-NHS. To produce a series of nanocomplexes with different sizes, OPSS-PEG-NHS with various lengths (2 K, 5 K, and 10 K) was used.

**Table 1:** Zeta potential measurements performed with bare Fe_3_O_4_-Au core-shell NPs, PEGylated NPs, nanobody-tagged NPs, and dye-labeled NPs at pH 7.4, presented as the mean of five runs ± SD

	Fe_**3**_O_**4**_-Au NPs		PEGylation	Anti-Muc1-Ab tagging	Cy3 labeling
**Zeta potential (ζ) (mV)**	− 19.8 ± 6.68	2 K	− 44.9 ± 8.19	− 38.5 ± 5.61	− 12.5 ± 7.25
		5 K	− 40.7 ± 7.88	− 23.3 ± 8.61	− 17.7 ± 3.94
		10 K	− 39.6 ± 8.74	− 31.8 ± 7.37	− 10.6 ± 4.72

After PEGylation, the zeta potentials were reduced (− 44.9 ± 8.19 mV, − 40.7 ± 7.88 mV, and − 39.6 ± 8.74 mV for 2 K, 5 K, and 10 K, respectively).

Interestingly, after nanobody tagging, the zeta potentials clearly increased (− 38.5 ± 5.61 mV, − 23.3 ± 8.61 mV, and − 31.8 ± 7.37 mV for 2 K, 5 K, and 10 K, respectively).

After dye tagging, the zeta potentials also increased (− 12.5 ± 7.25 mV, − 17.7 ± 3.94 mV, and − 10.6 ± 4.72 mV for 2, 5, and 10 K, respectively).

The zeta potential of bare Fe_3_O_4_-Au core-shell NPs was − 19.8 ± 6.68 mV. After PEGylation, the zeta potential was reduced to near − 40 mV. These results indicate that the PEG molecules were well bonded covalently to the Au shell of the core-shell NPs because PEG molecules have negatively charged N-hydroxysuccinimide functional groups. Meanwhile, the zeta potential increased after dye tagging to the nanobody (− 38.5 ± 5.61 mV for NP-PEG2 K-nanobody and − 12.5 ± 7.25 mV for NP-PEG2 K-nanobody-dye). This outcome is reasonable because the recombinant nanobody has ten lysine tails at the terminal end. Each type of nanobody was categorized by zeta potential measurement, and to determine antibody binding on the nanobody, we measured the fluorescence for each nanobody type.

**Fig. 3:**
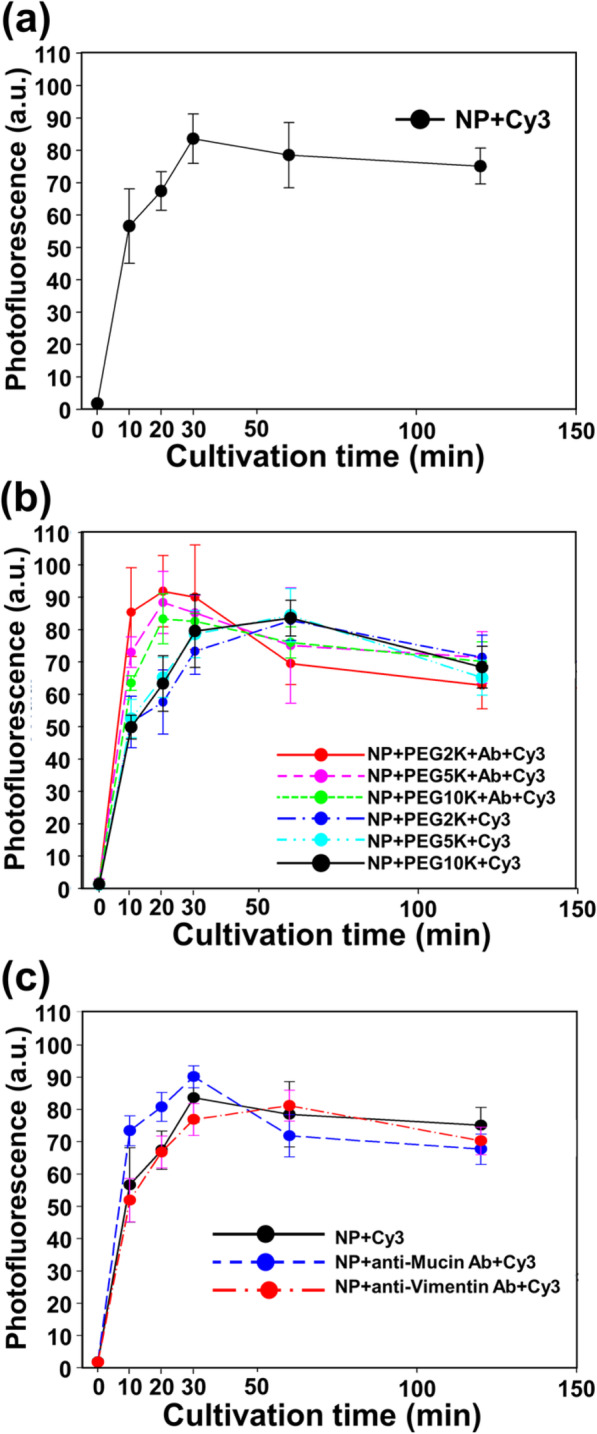
Normalized photofluorescence of CT26 mucin cells after incubation with 50 μg/mL **a** Fe_3_O_4_-Au NPs, **b** PEGylated NPs and PEG-nanobody-tagged NPs, and **c** Fe_3_O_4_-Au NPs, nanobody-tagged NPs, and vimentin-tagged NPs at 37 °C in 5% CO_2_ for different periods (10, 20, 30, 60, 120, and 360 min)

As shown in Fig. 3, we confirmed that all types of nanoparticles and nanobodies have well cellular uptake and internalization in the absence of inhibitor restrictions. A cellular internalization curve was obtained from cells incubated in the presence of 50 μg/mL Cy3-labeled Fe_3_O_4_-Au NPs, PEG-Cy3-labeled NPs, and PEG-nanobody-Cy3-labeled NPs for different periods (between 0 and 360 min) after removing media, washing out free NPs, and finally measuring the total fluorescence of the cells with trypan blue (Fig. 3).

According to the result of fluorescence intensity measurement, we can determine the NPs were internalized in the cells within 1 h (Fig. 3a). The fluorescence intensity of NP reached a maximum within 1 h, and the fluorescence intensity gradually decreased after reaching a steady state. Even though there is a slight time difference depending on the presence of Ab, the fluorescence intensity per cultivation time was not significantly different from the result of bare NPs (Fig. 3b). As shown in Fig. 3c, it was confirmed that the effect of Ab, by using heterogeneous Ab of the Muc1 and vimentin, was negligible in the cellular uptake and internalization of NP.

**Fig. 4:**
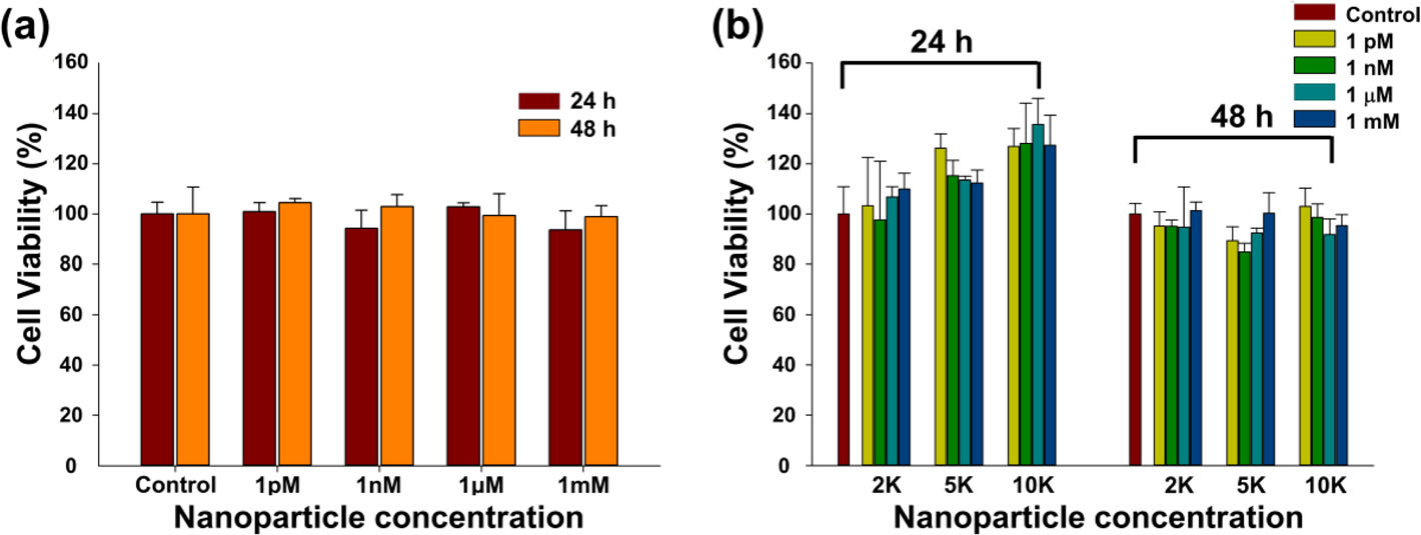
Viabilities of CT26 mucin cells treated with bare Fe_3_O_4_-Au core-shell NPs and surface-modified Fe_3_O_4_-Au NPs at different concentrations. **a** Fe_3_O_4_-Au NPs and **b** PEG-nanobody-Cy3-labeled NPs. Each experimental graph represents the average of a series of four different experiments

With WST-1 assay, the viability (%) of CT26 mucin cells depending on the concentration and surface modification of Fe_3_O_4_-Au core-shell NPs was estimated following various exposure times (Fig. 4a, b). The viability of CT26 cells did not show any significant differences following 24 h and 48 h of exposure, either with varying doses or after surface modification of the NPs. Cell viability was greater than 90% on both the bare Fe_3_O_4_-Au NPs (Fig. 4a) and the surface-modified NPs (Fig. 4b).

**Fig. 5:**
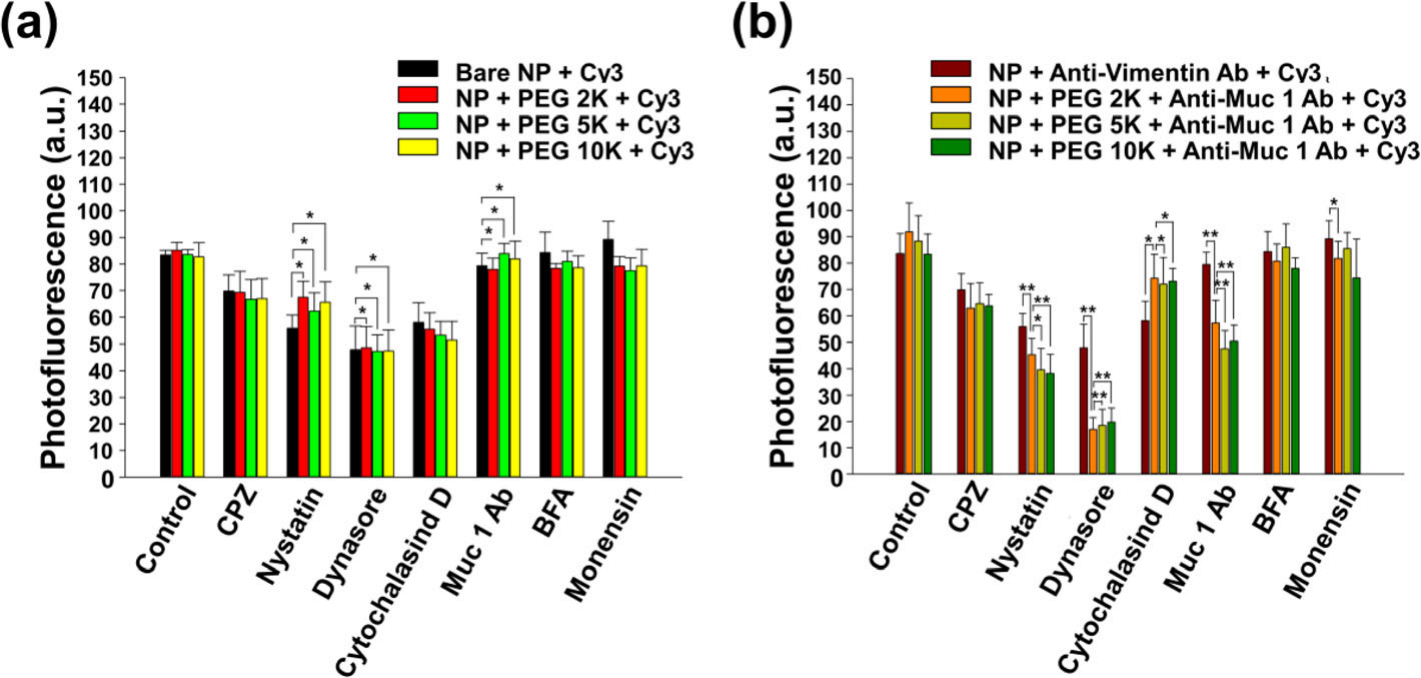
Normalized photofluorescence from CT26 mucin cells treated with chemical endocytosis inhibitors for 1 h and incubated with 50 μg/mL **a** bare core-shell NPs and **b** nanobody-tagged NPs at 37 °C in 5% CO_2_ for 30 min. Inhibitors with a statistical effect on the internalization (Student’s *t* test, *p* (*) < 0.05 and *p* (**) < 0.01) are marked with black asterisks

Figure 5 indicates that NPs entered the CT26 mucin cells via various endocytosis pathways (clathrin-mediated, caveolae-mediated, and phagocytosis/macropinocytosis pathways). Interestingly, Fig. 5b shows that anti-Muc1 Ab also mainly affected the endocytosis of PEG-nanobody-Cy3-labeled NPs. To understand the pathway of NP internalization, we tried to inhibit endocytosis pathways with specific chemical inhibitors (Fig. 5). The pathways of endocytosis were well known to be divided into three types: clathrin-mediated, caveolae-mediated, and macropinocytosis/phagocytosis.

In this study, inhibitors were used as a first approach to investigate the internalization of nanobody-tagged NPs. CPZ (clathrin-mediated endocytosis inhibitor), nystatin (caveolae-mediated endocytosis inhibitor), dynasore (dynamin inhibitor), cytochalasin D (phagocytosis/macropinocytosis inhibitor), BFA (Golgi apparatus destroyer), monensin (lysosome inhibitor), or anti-Muc1 Ab (receptor-/transporter-specific competitor) were incubated with cells for 1 h. CPZ, nystatin, dynasore, and cytochalasin D affected the endocytosis of NPs (Fig. 5a).

The targeting moiety is the key to the success of cancer-targeting, which is exceptionally important in cancer therapy. For targeting, effective surface modification is very important to increase therapeutic efficiency and limit side effects. Nanobody-tagged Fe_3_O_4_-Au core-shell NPs were successfully made from the synthesized NPs and recombinant nanobody. Table 1 clearly shows that each modification step was carried out successfully.

Cell viability is one of the essential elements for the biological application of nanomaterials. The cell viabilities were greater than 90% on the bare core-shell NPs and modified NPs (Fig. 4a, b). These results imply that bare Fe_3_O_4_-Au NPs and modified NPs did not cause significant concentration- and modification-dependent cytotoxicity and that the modified NPs were suitable for biological application.

The studies of internalization efficiency and the inhibitor effect of NPs provide important information to understand the mechanisms through which NPs enter cells. PEGylated NPs were relatively slow internalized into cells compared with bare NPs, but nanobody-tagged NPs were internalized into cells slightly faster than bare NPs (Fig. 3a, b). Because PEGylation is a well-known surface modification method to prevent the internalization of NPs, the internalization tendency of PEGylated NPs is easily explained. Moreover, the nanobody induced the endocytosis of NPs. To check the specificity of the nanobody, we confirmed the internalization rate of vimentin Ab-tagged NPs. Interestingly, vimentin Ab-tagged NPs did not promote cellular internalization (Fig. 3c).

These results indicate that the nanobody can effectively induce the internalization of nanomaterials into CT26 mucin cells and imply that the internalization tendency can be controlled with specific modification of the outer membrane of NPs. Additionally, the mechanisms of the endocytosis of nanobody-tagged NPs were clearly shown via inhibition tests and confocal microscopy imaging. The photofluorescence of both the nanobody-tagged NPs and the non-tagged NPs showed similar decreasing values when cultured with CPZ, nystatin, or dynasore (Fig. 5a, b). CPZ, nystatin, and dynasore play a role in inhibiting, respectively, clathrin-mediated endocytosis, caveolae-mediated endocytosis, and dynamin, which is a large GTPase implicated in the budding and scission of nascent vesicles from parent membranes. Thereby, the photofluorescence values in both cases decreased rapidly because dynamin is closely related to the production of vesicles for clathrin-mediated and caveolae-mediated endocytosis. As shown in Fig. 5a, both non-tagged (Fig. 5a) and nanobody-tagged NPs (Fig. 5b) showed rapid decreases in photofluorescence.

In particular, the nanobody-tagged NPs displayed significantly lower photofluorescence values in CPZ, nystatin, and dynasore. Moreover, we confirmed that the non-tagged NPs were more strongly affected than nanobody-tagged NPs were when applied to cytochalasin D, which is a cell-permeable toxin that blocks polymerization of actin filaments for phagocytosis [43]. These results imply that non-tagged NPs were internalized through multiple mechanisms such as clathrin-mediated endocytosis, caveolae-mediated endocytosis, and phagocytosis. Consequently, the cellular internalization of nanobody-tagged NPs depends on clathrin-mediated and caveolae-mediated endocytosis. Besides, the amount of cellular uptake of nanobody-tagged NPs was reduced considerably when cells were cultured with the Muc1 antibody (Fig. 5b). This result indicates that the free Muc1 antibody plays a role as a competitor of the nanobody on the modified NPs in attaching to the CT26 cell membrane and that the Muc1 antibody plays an important part in the cell internalization of the modified NPs. Peculiarly, vimentin Ab-tagged NPs showed obvious differences compared with nanobody-tagged NPs in terms of inhibition ability. The photofluorescence of vimentin-tagged NPs remained unaffected under multiple inhibition tests, indicating that the NPs were minimally influenced by nystatin, dynasore, cytochalasin D, and even the Muc1 Ab. This phenomenon could be evidence of the efficacy of vimentin, which has been biochemically confirmed to potently affect phagocytosis [39]. Consequently, this result indicates that the Muc1 Ab can target specific molecules and can control specific endocytosis.

**Fig. 6:**
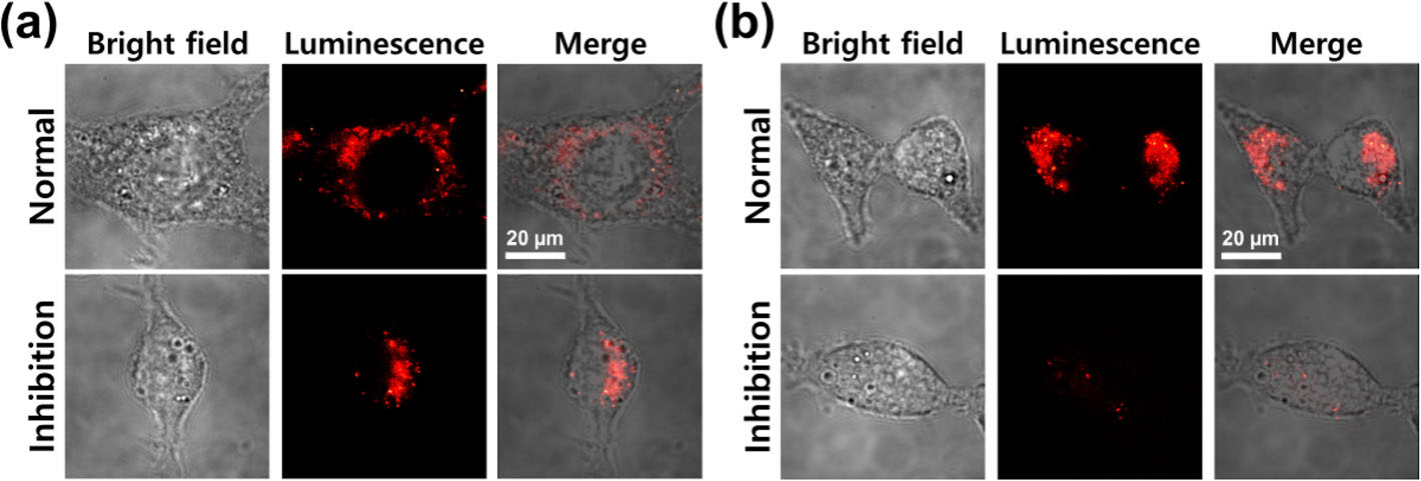
Confocal microscopic imaging of CT26 mucin cells incubated with **a** Fe_3_O_4_-Au NPs and **b** PEG-nanobody-tagged NPs for 1 h at 37 °C in 5% CO_2_ incubator, before and after dynasore inhibition (1 ng/mL)

As shown in Fig. 6, analogous results were obtained when the cells were treated with Cy7.5-labeled bare core-shell NPs and PEG-nanobody-tagged NPs, indicating similar cellular uptake in both cases in the absence of dynasore inhibition. Dynasore inhibition induced distinctly lower cellular internalization of the PEG-nanobody-tagged NPs compared with bare NPs (Fig. 6b, bottom row). These results imply that there are two endocytosis mechanisms, which are non-specific endocytosis of bare NPs and restricted endocytosis of nanobody-tagged NPs via the dynamin molecule. Once the nanobody attaches to the external cell membrane, the nanobody-tagged NPs could easily pass through the cell membrane because of the simultaneous activation of the clathrin- and caveolae-mediated mechanisms. Consequently, we could suppose that the main mechanisms are both clathrin- and caveolae-mediated endocytosis for the internalization of nanobody-tagged NPs in CT26 mucin cells.

## Conclusions

Nanomaterials for cancer targeting and controllable insertion of exogenic materials such as drugs, genes, and peptides are critical advances in biomedical applications. These familiar but creative concepts can offer strategies for new therapeutic methods. In this paper, we demonstrated enhanced cellular uptake of Fe_3_O_4_-Au core-shell NPs after PEGylation with the Muc1 antibody. The main endocytosis mechanisms of nanobody-tagged NPs were demonstrated, showing the possibility of controllable specific endocytosis in colorectal cancer cells. These findings provide insight into the targeting between nanobody-tagged NPs and colorectal cancer cells to aid the design of high-efficiency targeting carriers.

## Supplementary information

**Additional file1.** Supplementary information: Supplementary information accompanies this paper at https://doi.org/10.1186/s11671-020-03395-w. 1. Anti-MUC1-VHH 5-24 10 K ver.; 2. Characterization of core-shell Fe_3_O_4_-Au NPs and nanobody- Fe_3_O_4_-Au NPs; 3. Confocal microscopy imaging; 4. Cell viability test (WST-1 assay)

## Data Availability

All data generated or analyzed during this study are included in this published article.

## Abbreviations

Fe_3_O_4_: Magnetite
Au: Gold
NPs: Nanoparticles
Ab: Antibody
HCAbs: Heavy-chain antibodies
VHH: VH of the camelid HCAb
PEG: Poly(ethylene glycol)
PPG: Poly(propylene glycol)
PCR: Polymerase chain reaction
TEM: Transmission electron microscopy
PEO-PPO-PEO: Poly(ethylene oxide)-poly(propylene oxide)-poly(ethylene oxide)
OPSS-PEG-NHS: Orthopyridyl disulfide PDP PEG succinimidyl ester
BFA: Brefeldin A
CPZ: Chlorpromazine
