# Supplementary material for: Assessment of Cellular Uptake Efficiency According to Multiple Inhibitors of Fe3O4-Au Core-Shell Nanoparticles: Possibility to Control Specific Endocytosis in Colorectal Cancer Cells
Source: Nanoscale Res Lett. 2020 Aug 17;15:165. doi: 10.1186/s11671-020-03395-w (PMC7431494; doi:10.1186/s11671-020-03395-w)
Supplement: Supplementary file 1 — Additional file1. Supplementary information: Supplementary information accompanies this paper at https://doi.org/10.1186/s11671-020-03395-w. 1. Anti-MUC1-VHH 5-24 10 K ver.; 2. Characterization of core-shell Fe3O4-Au NPs and nanobody- Fe3O4-Au NPs; 3. Confocal microscopy imaging; 4. Cell viability test (WST-1 assay) [file 11671_2020_3395_MOESM1_ESM.docx]

Supplementary information

Assessment of cellular uptake efficiency according to multiple inhibitors of Fe_3_O_4_-Au core-shell nanoparticles: possibility to control specific endocytosis in colorectal cancer cells

Bo Gi Park^1†^, Yu Jin Kim^2†^, Ji hyun Min^3^, Taek-Chin Cheong^4^, Sang Hwan Nam^5^, Nam-Hyuk Cho^4^, Young Keun Kim^3*^, Kyu Back Lee^1*^

^1^ Department of Biomedical Engineering, College of Health Science, Korea University, Seoul 02841, Korea

^2^ Institute for High Technology Materials and Devices, College of Engineering, Korea University, Seoul 02841, Korea

^3^ Department of Materials Science and Engineering, College of Engineering, Korea University Seoul 02841, Korea

^4^ Department of Microbiology and Immunology, College of Medicine, Seoul National University, Seoul 03080, Korea.

^5^ Korea Research Institute of Chemical Technology, Daejeon 34114, Korea

^†^Bo Gi Park and Yu Jin Kim contributed equally to this work.

*Correspondence: ykim97@korea.ac.kr, kblee@korea.ac.kr

1. High-resolution TEM image of a Fe_3_O_4_-Au core-shell NP

Fig. S1 shows the the HRTEM image of a Fe_3_O_4_-Au core-shell NP. A fast Fourier transform (FFT) analysis of the HRTEM image was carried out assessing the spot diffraction patterns of both Fe_3_O_4_ and Au phases. Data analysis indicates that some spots correspond to the (400) atomic planes of the inverse spinel Fe_3_O_4_ phase (ICDD card no. 00-019-0629) with interplanar distances of 2.09 Å. Besides, some other spots correspond to the (111) and (222) atomic planes of the cubic phase of Au (ICDD card no. 00-004-0784) with interplanar distances of 2.35 Å and 1.17 Å, respectively.


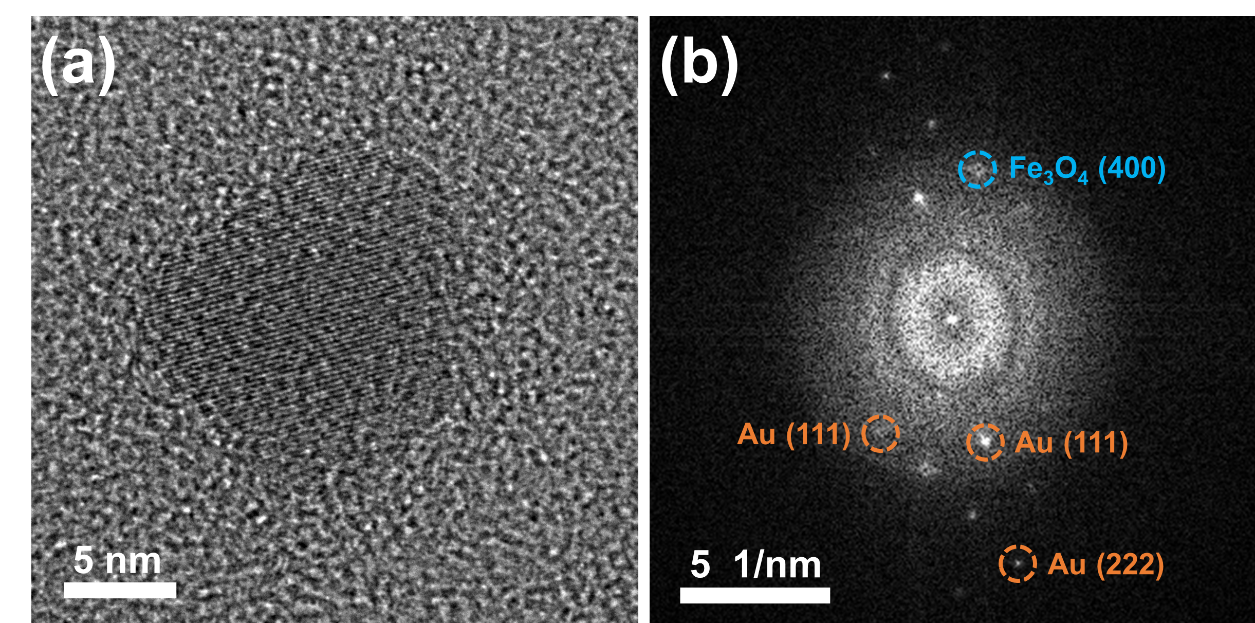


**Fig. S1** HRTEM image of an individual Fe_3_O_4_–Au core-shell nanoparticle. (a) HRTEM image of Fe_3_O_4_-Au core-shell NP, (b) corresponding FFT pattern acquired from the whole HRTEM image of Fe_3_O_4_-Au core-shell NP showing superlattice reflections Fe_3_O_4_ (400), Au (111) and Au (222).

2. EDX analysis of Fe_3_O_4_-Au core-shell NPs


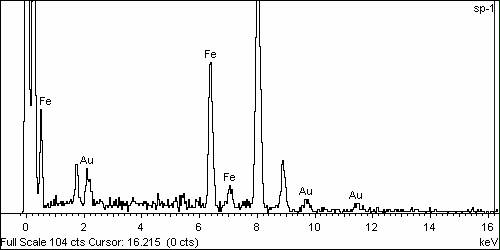


**Fig. S2** The Fe_3_O_4_-Au core-shell nanoparticle was characterized by the integration of the EDX-signal of specified regions. As a result of EDX analysis in the central region of the core-shell nanoparticles, it was confirmed that the iron core and gold shell signals were detected.

3. Anti-MUC1-VHH 5-24 10 K ver.

ATGGCTAGCATGACTGGTGGACAGCAAATGGGTCGCGGATCCGaattcGCCGATGTGCAGCTAGTGGAGTCTGGTGGAGGCTCGGTGGAGACTTTTGGATCTCTGAGGCTGTCGTGTGTTGCCACTGGTGACACCGTCGGTAGTGTTAGTATGGGTTGGTTCCGTCAGGCTCCAGGAAAGCCCCTTGAAGGAGTCGCTGCGTATAATCGCGCTGGTGGTGTCAGAGCCTACGCCGACTCCGTCAGGGGCCGATTCTCGGTTTCTCAAGAATACCGTTCCAATACACAGTATCTGAACATGATAAATCTGAGACCTGAGGACACGGGCATCTACTACTGTGCGGCAGGGCCCCACGACACCTTGGCACGTAATGGCAATCTTCGTCCAGATGGGTATATATACTGGGGCCAGGGGACCCAGGTCACCGTCTCCTCAGGCAAGAAGAAGAAGAAGAAGAAGAAGAAGAAGCTCGAGCACCACCACCACCACCACTGA

MASMTGGQQMGRGSEFADVQLVESGGGSVETFGSLRLSCVATGDTVGSVSMGWFRQAPGKPLEGVAAYNRAGGVRAYADSVRGRFSVSQEYRSNTQYLNMINLRPEDTGIYYCAAGPHDTLARNGNLRPDGYIYWGQGTQVTVSSGKKKKKKKKKKLEHHHHHH*

4. Characterization of core-shell Fe_3_O_4_-Au NPs and nanobody-Fe_3_O_4_-Au NPs

The morphology, size, and size distribution of the core-shell NPs were characterized by transmission electron microscopy (TEM, JEOL-2010F). Samples for TEM analysis were prepared as follows. After diluting the NPs in hexane, a drop of the diluted solution was placed on a carbon-coated Cu grid. Optical properties were analyzed by UV spectrometry (Shimadzu RF-5300 PC). To investigate optical absorption, the samples were diluted in water and measured at room temperature. The hysteresis curves of the core-shell NPs were obtained from vibrating sample measurements (VSMs, EV9-380V, Microsense). The VSMs were performed using dried samples on a piece of glass at room temperature. Using the core-shell NPs before and after PEG surface-modification in aqueous solution, the surface charge of the NPs was analyzed by a Zeta Potential Analyzer (Malvern, Nano-ZS 90). Samples for the zeta potential measurement were prepared by diluting aliquots of the original sample, and measurements were carried out at room temperature.

5. Confocal microscopy imaging

Cells (5 × 10^3^) were seeded on each confocal slide glass in six wells and incubated in 250 μL of medium for 24 h at 37°C and 5% CO_2_. The medium was removed, 250 μL of fresh culture medium containing 25 μg/mL dynasore was added, and the cells were incubated for 1 h. The medium was removed again, and 250 μL of culture medium containing 10 ng/mL Cy7.5-labeled Fe_3_O_4_-Au NPs, or PEG-nanobody-Cy7.5-labeled NPs were added. After 30 min at 37°C and 5% CO_2_, the cells were washed three times with medium to remove free NPs, and the fluorescence was measured at 785 nm.

6. Cell viability test (WST-1 assay)

Au-coated NPs are traditionally considered relatively nontoxic. However, many factors such as NP composition and concentration, cell line, and culture time affect cell viability. To assess the cytotoxicity of bare Fe_3_O_4_-Au NPs and nanobody-tagged NPs on CT26 mucin cells, we performed the WST-1 assay by incubating CT26 mucin cells with NPs at 1 pg/mL, 1 ng/mL, 1 μg/mL, and 1 mg/mL NPs for 24 h and 48 h.
